# An Electronically Delivered Person-Centered Narrative Intervention for Persons Receiving Palliative Care: Protocol for a Mixed Methods Study

**DOI:** 10.2196/41787

**Published:** 2023-03-21

**Authors:** Heather Coats, Nadia Shive, Bonnie Adrian, Andrew D Boyd, Ardith Z Doorenbos, Sarah J Schmiege

**Affiliations:** 1 College of Nursing University of Colorado Anschutz Medical Campus Aurora, CO United States; 2 UCHealth Aurora, CO United States; 3 University of Illinois Chicago, IL United States; 4 UI Health Chicago, IL United States; 5 University of Illinois Cancer Center Chicago, IL United States; 6 Department of Biostatistics and Informatics Colorado School of Public Health University of Colorado Anschutz Medical Campus Aurora, CO United States

**Keywords:** electronic health record, mixed methods, narrative, person-centered care, palliative care

## Abstract

**Background:**

In the health care setting, electronic health records (EHRs) are one of the primary modes of communication about patients, but most of this information is clinician centered. There is a need to consider the patient as a person and integrate their perspectives into their health record. Incorporating a patient’s narrative into the EHR provides an opportunity to communicate patients’ cultural values and beliefs to the health care team and has the potential to improve patient-clinician communication. This paper describes the protocol to evaluate the integration of an adapted person-centered narrative intervention (PCNI). This adaptation builds on our previous research centered on the implementation of PCNIs. The adaptation for this study includes an all-electronic delivery of a PCNI in an outpatient clinical setting.

**Objective:**

This research protocol aims to evaluate the feasibility, usability, and effects of the all-electronic delivery of a PCNI in an outpatient setting on patient-reported outcomes. The first objective of this study is to identify the barriers and facilitators of an internet-based–delivered PCNI from the perspectives of persons living with serious illness and their clinicians. The second objective is to conduct acceptability, usability, and intervention fidelity testing to determine the essential requirements for the EHR integration of an internet-based–delivered PCNI. The third objective is to test the feasibility of the PCNI in an outpatient clinic setting.

**Methods:**

Using a mixed method design, this single-arm intervention feasibility study was delivered over approximately 3 to 4 weeks. Patient participant recruitment was conducted via screening outpatient palliative care clinic schedules weekly for upcoming new palliative care patient visits and then emailing potential patient participants to notify them about the study. The PCNI was delivered via email and Zoom app. Patient-reported outcome measures were completed by patient participants at baseline, 24 to 48 hours after PCNI, and after the initial palliative care clinic visit, approximately 1 month after baseline. Inclusion criteria included having the capacity to give consent and having an upcoming initial outpatient palliative care clinic visit.

**Results:**

The recruitment of participants began in April 2021. A total of 189 potential patient participants were approached via email, and 20 patient participants were enrolled, with data having been collected from May 2021 to September 2022. A total of 7 clinician participants were enrolled, with a total of 3 clinician exit interviews and 1 focus group (n=5), which was conducted in October 2022. Data analysis is expected to be completed by the end of June 2023.

**Conclusions:**

The findings from this study, combined with those from other PCNI studies conducted in acute care settings, have the potential to influence clinical practices and policies and provide innovative avenues to integrate more person-centered care delivery.

**International Registered Report Identifier (IRRID):**

DERR1-10.2196/41787

## Introduction

### Background and Rationale

Health care advances have extended the life span and cured many diseases; however, advanced health care treatments are sometimes discordant with patient preferences, values, and beliefs, which can lead to insufficient symptom control, difficult patient-clinician interactions, and poor psychosocial and spiritual support [1-3]. Furthermore, poor patient-clinician communication contributes to continued unwanted care. A comprehensive palliative care approach improves communication, leading to better quality of life (QoL) for patients [4-6]; however, discordant care continues in part because of knowledge gaps about patients’ psychological, social, and spiritual needs [7-10]. Person-centered narrative interventions (PCNIs) can fill these knowledge gaps [11-20], yielding an increased understanding of patients’ psychological, social, and spiritual needs, which will help clinicians develop tailored palliative care interventions.

Storytelling (narrative) is an effective way for patients to communicate their cultural values and beliefs. As early as 1542, when Joannes Fernel coined the term physiology, the discussion at that time described the person as a structure of physiological, pathological, and clinical stories [21]. However, in the health care system, patients do not always have the opportunity to share their clinical stories with their clinicians [3,22-26]. Furthermore, even if patients have the opportunity to discuss values, beliefs, and preferences for care, some research has described treatment errors that may occur when clinicians overlook contextual information in a clinic visit. These treatment errors are known as contextual errors [27-29] and are defined as a “decision making error that occurs because of inattention to patient context” [29].

In the health care setting, the electronic health record (EHR) is one of the primary ways of communicating the status of patients; however, most of this EHR information is clinician centered. There is a need to consider the patient as a person and integrate their perspectives into their health record. Incorporating a patient’s narrative into the EHR provides an opportunity to communicate patients’ cultural values and beliefs to the health care team and has the potential to improve patient-clinician communication [30,31]. However, research on the effective integration of a patient’s narrative into the EHR is limited. PCNIs that incorporate cultural values and beliefs are important components of person-centered care [14,31]. Person-centered care includes implementing shared decision-making, providing holistic patient care with respect for patients’ preferences and goals, paying attention to nonmedical aspects of care, facilitating communication, knowing the patient as a person, and understanding culture, which influences health behaviors and the meaning of illness [3,14,22-26]. Quality communication is a critical element of person-centered care [3,32,33]. Story theory can be applied to person-centered care because of its foundation in “meaning making” built on promoting intentional dialogue, creating ease, and connecting with self-in-relation [34]. Using the foundation of story theory, patient narratives about illness experiences offer clinicians a view of patients’ cultural attitudes about their illness and their psychological, social, and spiritual beliefs and values [14,31]. When a clinician knows more of the patient’s narrative, improved quality of communication may occur, which can create opportunities for culturally congruent care with the potential to improve QoL for persons living with serious illnesses [3,32,33]. The PCNI is an example of how story theory might be applied to the fragmented health care system. However, in the current health care environment, limitations on a clinician’s time prevent these opportunities to better understand what may or may not improve QoL for persons living with serious illness. A narrative intervention implemented with persons living with serious illnesses can draw together the complexities and varied cultural meanings of illness experiences, and this can improve QoL [3,14,32,33]. Patient participation in narrative interventions has been linked to reduced pain, stress, anxiety, and fatigue and improved psychosocial and QoL outcome measures [15-20].

The integration of the PCNI into the EHR could benefit patients and clinicians interacting in technology-rich environments. Because the EHR is one of the primary modes of communicating health care information about the patient, the integration of patients’ narratives into the EHR has the potential to (1) improve person-centered care by incorporating patients’ values and beliefs, (2) provide opportunities to enhance patient-clinician communication, and (3) positively impact patients’ psychosocial and spiritual well-being. Minimal research has integrated narrative interventions into the EHR in a meaningful and efficient way to test whether the narrative intervention could improve communication between patients and clinicians and impact patients’ overall well-being. Our research program investigates innovative ways to integrate patient values, beliefs, and preferences into EHR through PCNIs [14,31]. A *central hypothesis* that guides our research is that the implementation of a PCNI will result in improved patient-clinician communication and care delivery and improve patients’ psychological and social well-being. We have conducted prior studies of PCNI in an acute care setting where the intervention was conducted in a face-to-face interaction [14,31]. However, owing to the COVID-19 pandemic, there is an urgent need to investigate innovative internet-based–delivered ways to incorporate the patient’s narrative into their health care record. In our fragmented health care systems, the lack of personhood in delivery of care has been linked to decreased QoL for persons living with serious illness [32,34-38].

### Study Context

We developed a PCNI to address existing gaps in the delivery of person-centered palliative care**.** Our first study on this PCNI used a narrative analysis methodology to elicit illness narratives to aid in understanding patients’ psychological, social, and spiritual experiences [37]. This led to a crucial clinical question: how might these person-centered narratives be shared efficiently and effectively with the health care team? Although the use of narratives is an effective way for people to communicate cultural values and beliefs [3,32,33], patients rarely have the opportunity to share such information with their clinicians [4,7,39,40]. Within the health care system, EHR is the primary mode of communicating patient information [30,31]. Thus, incorporating a person-centered narrative into the EHR is an opportunity to share patients’ values, beliefs, and preferences, with the potential to improve patient-clinician communication and well-being [14,31,37]. Our second study, a feasibility study, used the Consolidated Framework for Implementation Research (CFIR) [41] to define and refine the integration of the in-person delivery of PCNI into the EHR for persons with serious illness in the inpatient acute care setting [14]. Findings from the second study showed that the PCNI was (1) feasible and acceptable for all 20 hospitalized persons and (2) usable by acute care nurses [14]. Implementation data from our initial feasibility study revealed the ways in which PCNI is valuable and usable for clinicians. The PCNI’s added value in nursing care is when the nurse manager of one of the clinical units began reading the person-centered narratives aloud to her entire staff at shift handoffs. We also found value added for the entire health care team when 1 of the person-centered narratives was read during an ethics consultation. The ethics consultation was initiated owing to patient noncompliance and stated the desire to leave treatment against medical advice. Once they heard the person-centered narrative read during the consultation, the health care team realized why the patient was trying to leave before the proposed surgery—he needed to see his 2 daughters, who lived in another state, beforehand. We are currently conducting a third study, a 3-year mixed methods pilot study in the acute care setting [31]. Our long-term goal is to optimize the use of PCNIs by investigating how and in what context they are effective in improving patients’ overall well-being and quality of communication with their clinician. A challenge for any biobehavioral intervention is translation across complex health care contexts [42]. Because our in-person–delivered PCNI has been shown to be feasible and to demonstrate preliminary efficacy in the acute care setting, we aim to purposefully enroll participants in the outpatient setting and use an all-internet-based delivery of the PCNI to further evaluate implementation in a new setting and use an all-internet-based delivery—from email recruitment, electronic consent, and Zoom narrative interview to electronically delivered outcome measures.

### Objectives

In this protocol, we aim to further develop the continued testing of the feasibility and efficacy of the EHR integration of PCNI in an outpatient setting using an all-internet-based–delivered platform. The first objective of this study is to identify barriers and facilitators of an internet-based–delivered PCNI from the perspectives of persons living with serious illness and their clinicians. The second objective is to conduct acceptability, usability, and intervention fidelity testing to determine the essential requirements for the EHR integration of an internet-based–delivered PCNI. The third objective is to test the feasibility of the PCNI in an outpatient clinic setting.

## Methods

### Study Design

This study will use a mixed methods design [41,43-45] and collect data from both patient and clinician participants. Our study relies on the CFIR [41,45] to inform the implementation of this single-arm trial across the 2 years of the study. This is a novel approach that documents what affects implementation in a new context of outpatient clinical care with a protocol that is all delivered electronically. The CFIR is a conceptual framework developed to guide multilevel assessment of factors that may influence an intervention’s implementation and effectiveness [41,45]. The core CFIR domains are (1) intervention characteristics, (2) inner setting (ie, implementing organization), (3) outer setting (ie, external environment), (4) individual characteristics (ie, knowledge and beliefs of the individuals involved in the implementation), and (5) processes (ie, strategies and tactics used in the implementation [41,45]). Within each domain, specific constructs may influence the implementation. Constructs in the proposed taxonomy of implementation outcomes have the potential to capture clinician attitudes (acceptability) and behaviors (adoption and uptake) as well as contextual factors (system penetration, appropriateness, and implementation).

In this phase of our biobehavioral interventional development, both quantitative and qualitative data give necessary information about acceptability, feasibility, and usability for the optimization of the narrative intervention. Quantitative data include longitudinally measured patient-reported outcomes, and qualitative data include (1) patient and clinician exit interviews for triangulation of the quantitative findings and (2) patients’ illness narratives. All data collection and study procedures will be conducted electronically, using secure videoconference, phone, REDCap (Vanderbilt University) [46] surveys, and electronic consent forms. Evaluating the barriers to and facilitators of the implementation of the adapted internet-based delivery of PCNIs is essential for effective translation to practice. Such a design is particularly salient when conducting studies with a palliative care population [47-49] to collect a broad range of data. For example, our qualitative data will provide a deeper understanding of the barriers to and facilitators of the intervention from participants’ perspectives not captured through the traditional quantitative measures [50-52]. We will analyze key implementation facilitators and challenges and link these to the outcomes [51]. This work will result in an evidence-based set of core functions and forms of this complex intervention into complex health care systems by providing recommendations to guide future PCNI implementation across different health care settings [53-55].

### Participants

Purposeful sampling strategies will be used to recruit and enroll 20 patients. The small sample size was chosen to be consistent with both qualitative data analysis techniques [50] and usability data analysis techniques [56,57]. Patient participants (N=20) who are aged ≥18 years (no upper limit), able to read and speak English, and capable of providing informed consent will be enrolled in the study. Owing to the feasibility of this study, we allowed participants to enroll at any stage of illness or symptom acuity. These participants will be newly establishing care at one of the University of Colorado Hospital (UCHealth) outpatient palliative care clinics (Outpatient Palliative Care Clinic and Supportive and Neuro-Palliative Medicine Clinic). The clinician participants (N=10) will be members of the outpatient palliative care interprofessional team, which includes physicians, advanced practice clinicians, registered clinicians, social workers, and a spiritual care clinician. These clinician participants will be included if they are able to confirm verbally that they were involved in the outpatient palliative care of a patient participant.

For the patient participants, the research team will review the outpatient palliative care clinic schedules weekly via the EHR (Epic) to identify persons with upcoming initial new patient visits (NPVs) in the palliative care clinics. These identified persons will receive an initial recruitment email with an institutional review board–approved language and a follow-up email with the initial language approximately 1 week later for those who have not responded. For those who respond to the recruitment email with interest, the research team will arrange a secure videoconference or phone call to discuss the study, review the consent form, and answer questions. When a person opts to participate, the research team will send an email with a link to the electronic consent form (in REDCap) [46] and instructions for completing the consent. It should be noted that persons are contacted approximately 4 weeks before their NPV to facilitate ample time for pre-NPV study activities.

For the clinician participants, the research team will identify the assigned clinician of the enrolled patient participant, once the patient’s narrative is uploaded to the EHR. In the same electronic delivery, the research team will contact these clinicians via email to (1) alert them that a narrative was available for one of their newly establishing patients and (2) inquire about their interest in participating in the study using institutional review board–approved language. Because of the nature of the feasibility and usability study, we targeted recruitment efforts to capture the entire interprofessional perspectives of the palliative care clinical team members. For those who respond to the recruitment email with interest, a research team member will arrange a secure videoconference or phone call to discuss the study, review the consent form, and answer questions. When a clinician opts to participate, the research team will send an email with a link to the electronic consent form (in REDCap) [46] and instructions for completing it.

### Procedures

#### Ethics Approval and Data Privacy

All procedures were approved by The University of Colorado Institutional Review Board (Colorado Multiple Institutional Review Board 21-2887), and the approval has been maintained in good standing. REDCap is available as a Health Insurance Portability and Accountability Act–compliant secure research data interface [46]. Web servers routinely use secure socket layer encryption technology and certification authority to ensure the secure transmission of data over the internet. To facilitate secure file sharing between research team members, we will establish internet-based private networks and create sponsored user accounts for all study personnel. All research data files are stored on dedicated, password-protected, access-controlled, and shared drives. Safeguards for protecting participants’ anonymity will include creating a master list of names with corresponding numbers assigned to participants at the time of the enrollment. Once assigned, the corresponding numbers will then be used to deidentify the remainder of the participants’ personal information and each participant’s data. Electronic consent forms, participant identity, and participant data will be securely stored on a password-protected network server. Participants will only be identifiable by an ID number on the server, and only anonymized data will be provided to the data analysis team members. The potential for breach of confidentiality is being addressed through the maintenance of a secure “log-on” system on the secure computer server dedicated to this protocol.

All participants will be compensated for their time. Patient participants will be provided a US $25 electronic gift card after the completion of PCNI and time point 2 and a second US $25 gift card after the completion of time point 3 for a total of US $50 in remuneration. Clinician participants will be provided one US $25 electronic gift card after the completion of the exit interview. In addition to the confidentiality of patient data, there are some minimal physical, social, or psychological risks to the patient participants. Participants could become fatigued during the 60-to-90–minute narrative interview process, the 30-minute exit interview process, outcome measure data collection procedures. In the event of fatigue, the research team members will encourage rest periods or, if necessary, schedule an additional study visit to complete the interview. For the patient participants, there is a minor, moderate, or severe risk of psychological distress because of the sensitive topics of serious illness. Because of the feasibility of the design, we will ensure that the patient participants understand both orally and in writing that they are free to decline participation in any or all study activities at any time based on their own level of distress [58,59]. This could include declining to answer specific questions, refusing participation in the intervention, requesting to speak off the record, or requiring complete withdrawal from the study. We did not apply a specific tool for distress screening; rather, we will give frequent reminders to the participants about the voluntary and autonomous nature of the study, for the participants to determine if the study activities are too distressing for them at any level (mild, moderate, or severe). If any of the interview or psychometric assessment data reveal increased participant distress, dissatisfaction, or any adverse event, the data collection will cease. If the participant exhibits any verbal or nonverbal distress, the research team member will allow the participant to stop the interview, reschedule for a return visit to complete the interview, or allow participants to withdraw consent. If moderate distress is exhibited, the research team member will assist in referring participants to emotional support through family, friends, or the health care team for the follow-up related to the participant’s distress. If distress is severe, such as suicidal ideation, the research team member will immediately refer the patient for further work-up and care. The research team members will carry a list of contact phone numbers for such referrals. If any moderate or severe distress is noted, it will be reported immediately to the principal investigator to help determine if the event meets the criteria for a reportable adverse event. If deemed a serious adverse event, the institutional review board will be notified, and the participant’s involvement in the research study will be discontinued. In a previous study using this methodology, none of the adverse events occurred. Therefore, we anticipate these risks to be rare events, given the nature of the data collection procedures.

#### The PCNI Procedure

**Figure 1:**
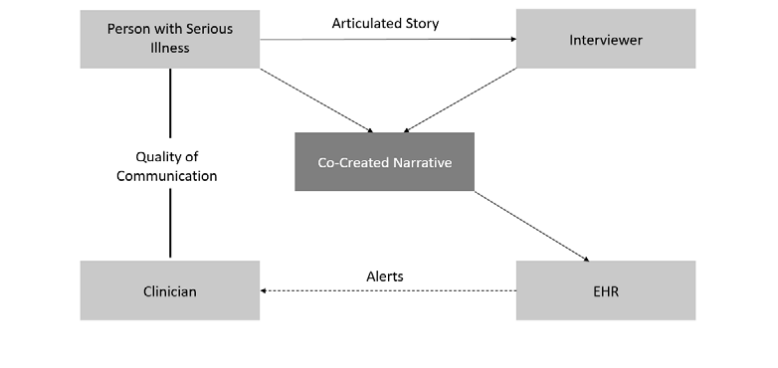
Person-centered narrative intervention (PCNI) workflow figure. EHR: electronic health record.

The enrolled patient participants will participate in a narrative interview. These audio-recorded interviews will be conducted by a research team member using an open-ended interview guide. During the interview, patients will be prompted to share their narrative through probing questions or statements such as: tell me about your illness; tell me how your illness has affected your emotions, your relationships, and your spirituality (Textbox 1 lists the interview questions). These probing questions have been field tested in 3 previous studies. As the patient responds, the research team member will take field notes and ask for follow-up or clarification questions as necessary. Using the audio recording and field notes, the research team member will then create a 1-to-3–page metanarrative. Each narrative must meet the following criteria: it must (1) be written in the patient’s first-person voice, (2) be nonjudgmental, (3) capture the patient’s voice, (4) accurately reflect the content of the interview, and (5) nondiagnostic (not labeling). The patient narrative is then returned to the patient within 48 hours for member checking, the cocreation phase of the PCNI. In this electronically delivered protocol, at the time of member checking, the patient will make any desired changes to the narrative on their own or during a follow-up session with the research team member. These sessions will facilitate the cocreation of the narrative. Once the narrative is approved by the patient, the research team will upload it to the patient’s EHR and alert the patient’s assigned clinician via email that their patient’s narrative is available in the patient’s health record. The workflow diagram is depicted in Figure 1.

**Interview guides for narrative and exit interviews.**

Narrative intervention interview guide

- Conversational questions
  - May I call you?
  - What is your illness?
- Conversational probes throughout intervention
  - For example
  - Tell me more about that
  - Anything else?
- Open-ended questions
  - Tell me what it has been like to have _____ (illness).
  - Tell me how your illness has impacted you emotionally, your feelings?
  - Tell me how your illness has impacted your relationships with family, friends, and others?
  - Tell me how your illness has impacted your spirituality? Your faith, beliefs, your values, or your thoughts about a higher power?
- Closing question
  - Is there anything else we have not talked about that you would like to tell me?

Exit interview guide: patient participants

- Describe what you liked about participating in this study.
- Describe what you did not like about participating in this study.
- Did you have the opportunity to discuss your story with anyone other than the research team?
  - *If yes, follow-up questions*
    - Who did you discuss it with?
    - Did you initiate the conversation, or did they?
    - Describe which parts of the story you discussed.
    - How did this discussion make you feel?
  - *If no, follow-up questions*
    - Why do you think others did not discuss it with you?
    - Why did not you discuss it with others?
- Is there anything you would have changed about the study?
- What would you think about participating in a similar intervention in the future?
- Is there anything else you would like us to know?

Exit interview guide: clinician participants

- What did you think about the intervention?
- Were you able to read the patient’s story?
- Do you think the study was beneficial to the patients?
- Did you notice any particular patient reactions to the intervention?
- Was the study beneficial to you in any way?
  - *If yes, follow-up questions*
    - Describe what was beneficial to you.
  - If no, follow-up questions
    - Describe what was not beneficial to you.
    - What changes could we make to the study for it to be more beneficial to you?
- Do you feel like having the patient’s story changed how you delivered care to your patient?
  - *If yes, follow-up questions*
    - Can you provide an example?
  - *If no, follow-up questions*
    - Are there changes we could make that would help with this?
- Did the notification system of the story in the medical record work for you?
  - *If no, follow-up questions*
    - How could the notification system be improved?
- Were there any other study difficulties that you encountered that I have not asked you about?
- Are there improvements to the intervention that you would suggest that we have not already discussed?
- Is there anything else you would like us to know?

#### Outcome Evaluations of the PCNI

##### Usability Testing

Using a human, organization, and technology–fit factor [53] evaluation framework for health information systems, we will collect data across several usability factors. Usability is defined as the relationship between humans and computers [53]. Usability testing [53,56,57,60] focuses on evaluating process measures based on 4 major components: user (clinician), task (use of metanarrative), system (metanarrative in EHR), and environment (outpatient setting). This user-task-system-environment framework of process measures [61] will be incorporated into our usability testing to determine barriers and facilitators from the clinician’s perspective, and to help determine the essential requirements for integrating the narrative intervention into the EHR by evaluating information flow, use of information, and system functionality. The process measures collected to determine usability will include (1) chart review and log analysis of the clinical use of the patient’s story; (2) time and cost analyses of collecting the story, writing and cocreating the metanarrative, and uploading the final metanarrative into the EHR; and (3) exit surveys and interviews with both patient and clinician participants.

##### Intervention Uptake and Attrition

We will also analyze the intervention uptake and attrition between baseline and follow-up data collection points [47-49]. Uptake (acceptability) will be assessed using descriptive summaries examining acceptors among people approached for recruitment. The number of dropouts, reasons given, and the timing of withdrawals from the study will also be reported. When possible, patient participants who drop out will be provided with a 1-page query about why they left the study. Feasibility will be examined by examining operational issues, namely, staff time spent or issues with recruitment, EHR use, or training. Identified issues will be noted in project logs to guide future studies. These are critical steps for evaluating scalability for the successful implementation of PCNIs in other complex systems. This approach incorporates the human, organization, and technology–fit [53] factors for the successful implementation of PCNIs.

#### Patient-Reported Outcomes

The primary patient-reported outcome measures for this protocol are the PROMIS (patient-reported outcomes measurement information system)-29 Profile v2.0 form [62] and PROMIS Psychosocial Illness Impact [63]. The PROMIS-29 profile v2.0 form consists of 29 items that assess physiological, social, and psychological outcomes. These biopsychosocial domains include physical function, anxiety, depression, fatigue, sleep, the ability to participate in social roles or activities, and pain interference and intensity. The PROMIS Psychosocial Illness Impact measure consists of 16 items that assess the negative and positive aspects of the illness experience. The PROMIS positive item bank measure (8 items) assesses the positive psychosocial outcomes of illness. Positive psychosocial illness impacts refer to outcomes, such as greater life appreciation, interpersonal relationships, and personal resources, that can occur because of confrontation with one’s mortality. The PROMIS negative item bank measure (8 items) assesses the direct negative psychosocial effect of illness, distinct from general emotional distress. All PROMIS measures have established reliability and validity. At baseline, demographic information will also be collected from patient participants, including age, biological sex, gender, race and ethnicity, perceived illness severity, years of education, income, marital status, religion or spirituality preference, and clinical details (to identify comorbidities and severity of illness).

The primary outcome measures for the patient participants will be collected across 3 time points. After enrollment, the research team will generate participant-specific survey links in REDCap [46] and email them to the participants to complete the demographic survey and the baseline (time point 1) outcome measures (Table 1). Outcome measures could also be collected by phone, if preferred by the participant. At a mutually scheduled time, the participant and a research team member will meet on a secure videoconference to conduct and audio record the PCNI interview, as described previously in *The PCNI Procedure*. Within 48 hours after the narrative is uploaded into the EHR, participants will complete time point 2 outcome measures using the same process described for time point 1. Time point 3 will be completed 1 month after the narrative upload and the NPV. Finally, after time point 3, the research team will conduct exit interviews with patient participants at the end of their study participation to collect information about their experience, including suggestions for improvement (Textbox 1 lists the interview questions).

For the clinician participants, the research team will conduct exit interviews with clinician participants within 2 to 4 weeks of their initial appointment with a patient participant to collect information about their experience of having and using the patient narrative before the initial consult. At the end of study enrollment, 1 to 2 audio-recorded focus groups (based on scheduling needs) will be cofacilitated by research team members with all the clinician participants to collect additional information and feedback about their experience and facilitate and record group discussion about their use of the patient narratives in their clinical practice. Interviews will be conducted via a secure videoconference or a phone call and audio recorded. See Table 1 for participant study activities.

**Table 1:** Participant study activities.

Participant	Outcome	T1: baseline (prenarrative upload)	T2: 24-48 hours after T1 (postnarrative upload to EHR^a^)	T3: 1 month after T1
Patient	Biopsychosocial and spiritual well-being	PROMIS^b^-29-Profile PROMIS Psychosocial Illness Impact; positive and negative effects	PROMIS-29-Profile PROMIS Psychosocial Illness Impact; positive and negative effects	PROMIS-29-Profile PROMIS Psychosocial Illness Impact; positive and negative effects
Patient clinician	N/A^c^	N/A	N/A	Exit interviews

^a^EHR: electronic health record.

^b^PROMIS: patient-reported outcomes measurement information system.

^c^N/A: not applicable.

### Data Analysis

#### Quantitative Analysis

Analyses will be performed using SAS software (version 9.4; SAS Institute Inc) [64]. Analyses will be primarily descriptive to determine the feasibility of collecting patient-reported outcome data in this setting (ie, data completeness) and summary statistics and distributional characteristics of those outcomes. Data will be inspected for errors and outliers. Data completeness will be summarized in terms of missing responses across the 3 data collection time points (ie, percent attrition at each follow-up) as well as missing item responses within each measurement period (ie, inspection of item-level missing data patterns). A high level of retention (>90%) across follow-up waves is expected based on previous research in an inpatient setting [14]; however, this will be empirically investigated. Missing responses at the item level are similarly expected to be low, but if present, they will provide information on potential response fatigue or problematic items. Baseline demographics and outcome measures at all 3 waves will be summarized using standard descriptive statistics (eg, mean, SD, and range). Preliminary testing of the outcome measures for change from baseline, T2, and T3 will be analyzed in a repeated measures framework (time effect in mixed effect model) to evaluate the effects of the intervention on the individual patient participant’s patient-reported outcomes of the PROMIS 29 Profile and PROMIS Psychosocial Illness Impact. This single-arm study is not powered to evaluate the statistical significance of changes in patient-reported outcomes over time; however, distributional characteristics of outcomes and effect size estimates of mean change will be useful for informing a larger-scale randomized trial.

#### Qualitative Analysis

Qualitative data management software will be used to organize narrative and exit interview data and coding by team members. Notes and transcripts will be collated to provide summaries of patient flow, use of equipment and supplies, perspectives on the PCNI, and ideas about its integration into current practice. A conventional content analysis approach [50-52] will be used to analyze the observations and interviews, and a category system will be created based on the CFIR domains to create a codebook [41,45] (with new codes added if needed). Obtaining data from a variety of interviewees allows for triangulation of data sources to support qualitative rigor. Triangulation of data will also occur between narrative interview data and exit interview surveys to better understand PCNI implementation fidelity and to identify any potential or needed modifications. The research team expects that the outcomes of this analysis will inform the next steps of PCNI integration as we move to implementation at future sites and for future studies. The information gained through this analysis will be used to provide a greater empathetic view of what is currently offered and what matters most to patients and clinicians, as they offer and integrate new evidence-based practices or interventions. We will extract contextual factors (events or statements) from observations (study notes) and interviews to document what facilitates or acts as a barrier to implementation (eg, challenges, resolutions, and the impacts of champions and naysayers). Quantitative data, from patient logs of session completion and the tracking database, will be integrated with qualitative data from observations and transcripts of semistructured interviews with patient participants and nurse participants. Triangulating our data sources will allow us to corroborate the evidence, prevent researcher bias in interpretation, and increase the confirmability of our qualitative data. The first round of coding will be guided by the CFIR domains and constructs [41,45], which include (1) intervention characteristics, (2) inner setting (ie, implementing organization), (3) outer setting (ie, external environment), (4) individual characteristics (ie, knowledge and beliefs of the individuals involved in the implementation), and (5) process (ie, strategies and tactics used in the implementation). Within each domain, specific constructs may influence the implementation. We will also capture emergent themes in the data (the inductive component) [43], which will allow for the discovery of themes not included in a priori CFIR codes. We will follow an iterative process, whereby the analysis will begin at the time of the first observation and will inform the direction and content of future data collection. Research team members will code the data independently and discuss it with other team members. To maximize convergence in coding [43], the team will meet regularly during data collection and analysis to review emerging themes, reconcile differences in coding, and determine whether modifications to the interview guide are needed for the remaining interviews. Discrepant interpretations [43] of the interview data during the coding phase will be resolved by consensus during team meetings. We will maintain an audit trail [50] to document all coding and analytic decisions made during the study. We will conduct between-case analysis to compare cases to look for similarities in processes promoting implementation among similar cases and differences in processes promoting sustainment among different cases [43]. We will analyze coded data by constructing causal diagrams that link the coded elements, logically minimizing the diagrams to produce a parsimonious set of pathways, and using explicit decision rules to guide the analysis [43,55].

## Results

The study received funding in January 2021, and ethics approval was received on March 21, 2021. The recruitment of participants began in April 2021. Patient participant recruitment was conducted via weekly screening of the outpatient palliative care clinic schedules for upcoming new palliative care patient visits, and emails were sent to potential patient participants. At this time, some data have been collected and analyzed. A total of 189 potential patient participants were approached via email, and 20 patient participants were enrolled, with data having been collected from May 2021 to September 2022. A total of 7 clinician participants were enrolled, with a total of 3 clinician exit interviews conducted in 2021 and 2022 and 1 focus group (n=5) conducted in October of 2022. Data analysis has been ongoing from the end of data collection in October 2022 to June 2023. Overall, for this feasibility study, we were able to recruit a total of 20 patient participants and 7 clinician participants for data collection. In the initial findings, the electronically delivered person-centered narrative was feasible, acceptable, and usable for both patient and clinician participants. The overall findings are expected to be submitted for publication by the end of June 2023.

## Discussion

### Overview

Although PCNI is not new [14,31], it has never been studied in an outpatient setting and completed via an all-electronic, internet-based–delivered protocol, a necessity to implement in our postpandemic world. Keeping the safety of all at the forefront in the setting of a continued global pandemic, it was necessary for the PCNI to be evaluated on an electronically and internet-based–delivered platform. In comparison with our previous studies on PCNI [14], the all-internet-based–delivered platform to conduct the study activities and collect person-centered narratives did have some challenges.

Using the CFIR as our framework [41,45], we encountered some implementation challenges. In the CFIR domain of the intervention, we did not change any components of the institutional review board–approved person-centered narrative interview conducted in the Zoom app (Zoom Video Communications Inc). We did not change anything from the domain of the inner setting. The entire study was conducted in the outpatient clinic population of persons with serious illnesses who were having an initial palliative care visit. It was feasible to cocreate the PCNI and upload the PCNI before all the initial palliative care visits of patients, with the exception of 1 patient participant. For future studies, there needs to be ample time between narrative interviews and a person’s initial visit to the palliative care team. Alternatively, narrative interviews could be conducted at any time of a person’s clinical care without the need for the narrative to be uploaded before a patient’s initial visit.

In contrast to our acute care studies [14], we did not realize the longer time required to complete the member checking of the cocreated narrative. This process took an average of 5 days versus an average of 48 hours in our acute care studies. This increased time links to the CFIR domain of the outer setting. Persons were not as acutely ill as in the acute care studies of the PCNI. The outpatient participants spent more personal time changing, adding, or adjusting their cocreated narratives. Therefore, the individual characteristics of this outpatient population were distinctly different from those of other patients who are hospitalized in our previous studies. Because these patient participants were also active in their home environments and not hospitalized, additional time was required to obtain the second Zoom app–facilitated session scheduled for the member-checking step of the PCNI review and the approval of their narrative before the narrative was uploaded into the EHR for the clinical team.

In the last CFIR domain of process, we implemented several strategies and tactics for the successful implementation. As we began email recruitment and electronic consenting, we chose not to enroll any patient participant who was noted to have a medical diagnosis of Alzheimer disease or dementia, or terms of cognitive impairment, confusion, or delirium on their problem lists. For this study, this screening excluded 20% of persons with a new patient palliative care visit. At this stage of data analysis, we would suggest that future PCNI programs should consider cognitive impairment. In future studies, the need for a mental capacity screening can be an important consideration. Alternatively, in future studies, the PCNI would need to consider the cognitive capacity of persons who have a serious illness and include a person’s support individuals to be a part of a dyadic PCNI. It should be noted that the integration of the PCNI into an outpatient palliative care clinic provides systematically more time for palliative care clinicians to engage with the narrative based on the allowed new palliative care patient visit time allotment of 1 hour. In future studies in other clinical settings that are nonpalliative, the acceptability and usability of the person’s narrative will require clinical workflow evaluations of the best strategies for allowing the nonpalliative clinician adequate time to engage with the person’s narrative. As in our other PCNI studies, once a patient participant is enrolled, there are limited withdrawals. Only 1 participant reported mild distress with the narrative interview questions that were being asked and made a choice to withdraw from the study.

Finally, although the overall goal of the PCNI is to consider the improvement of the quality of communication in clinical care, for this protocol, the quality of communication is being analyzed by exit interviews with both patient and clinician participants. In our other PCNI study [14], the quality of communication measure has been used [65]. However, in this study, the patient participants were enrolled before NPVs with the palliative clinical team. Therefore, we were unable to quantitatively measure any changes in the quality of communication between the patients and palliative care clinicians. For future studies of PCNIs, the consideration of not timing the PCNI before an NPV should be considered.

### Limitations

There are known limitations to our mixed methods single-intervention feasibility study. First, it was conducted in 1 geographic location in 1 health care system. This is an important consideration when considering the contextual complexities of health care systems, their EHRs, and the clinical workflows of clinicians. Second, owing to the nature of the internet-based delivery in this study, this study excluded participants who did not have access to email and technology with an internet connection. During the screening for this study, 11 patients did not have email addresses. Therefore, email should not be the only recruitment method. One would need to add an in-person recruitment approach to capture potential participants who do not have an EHR-listed email. During this in-person session in the clinical setting, one could also verify a person’s technology needs and preferences. If the preference or need for technology was identified, the opportunity to conduct the PCNI in person in the outpatient clinical setting could also be used. In this study, participants who expressed less agility with technology were offered more detailed instructions (“click-by-click”) or additional phone calls during which the research team member could help them navigate the required systems.

### Conclusions

This 2-year feasibility study will provide a site-specific understanding of barriers to and facilitators of the usability and acceptability of the PCNI from the perspectives of both patient and clinician participants. These barriers and facilitators in the setting of a CFIR framework will provide important knowledge about the scalability of the intervention, process-oriented insights, successful implementation strategies, and modifications that are necessary to improve the integration of a person’s narrative into the clinical workflow for clinicians. The overall long-term research goal is the creation of a PCNI that could be broadly applicable and sustainable outside of the research setting, with implementation in a variety of health care settings in multiple health systems. The knowledge gained will provide necessary information to evaluate future ways to scale PCNIs, with the goal of implementation across health systems, and will continue to inform the integration of person-centered narratives into the patient’s EHR. This feasibility study will provide important information to fully understand the mechanisms of the effects and concurrently contribute to additional knowledge of the key factors of implementation. The knowledge gained from this mixed methods study will contribute to key data for the continued development and refinement of PCNIs.

## Abbreviations

CFIR: Consolidated Framework for Implementation Research
EHR: electronic health record
NPV: new patient visit
PCNI: person-centered narrative intervention
PROMIS: patient-reported outcomes measurement information system
QoL: quality of life

## Data Availability

The data sets generated and analyzed during this study are not publicly available owing to patient participant confidentiality but are available from the corresponding author upon reasonable request.
